# Profiling of Small Nucleolar RNAs by Next Generation Sequencing: Potential New Players for Breast Cancer Prognosis

**DOI:** 10.1371/journal.pone.0162622

**Published:** 2016-09-15

**Authors:** Preethi Krishnan, Sunita Ghosh, Bo Wang, Mieke Heyns, Kathryn Graham, John R. Mackey, Olga Kovalchuk, Sambasivarao Damaraju

**Affiliations:** 1 Department of Laboratory Medicine and Pathology, University of Alberta, Edmonton, Alberta, Canada; 2 Department of Oncology, University of Alberta, Edmonton, Alberta, Canada; 3 Cross Cancer Institute, Alberta Health Services, Edmonton, Alberta, Canada; 4 Department of Biological Sciences, University of Lethbridge, Lethbridge, Alberta, Canada; National Institute of Technology Rourkela, INDIA

## Abstract

One of the most abundant, yet least explored, classes of RNA is the small nucleolar RNAs (snoRNAs), which are well known for their involvement in post-transcriptional modifications of other RNAs. Although snoRNAs were only considered to perform housekeeping functions for a long time, recent studies have highlighted their importance as regulators of gene expression and as diagnostic/prognostic markers. However, the prognostic potential of these RNAs has not been interrogated for breast cancer (BC). The objective of the current study was to identify snoRNAs as prognostic markers for BC. Small RNA sequencing (Illumina Genome Analyzer IIx) was performed for 104 BC cases and 11 normal breast tissues. Partek Genomics Suite was used for analyzing the sequencing files. Two independent and proven approaches were used to identify prognostic markers: case-control (CC) and case-only (CO). For both approaches, snoRNAs significant in the permutation test, following univariate Cox proportional hazards regression model were used for constructing risk scores. Risk scores were subsequently adjusted for potential confounders in a multivariate Cox model. For both approaches, thirteen snoRNAs were associated with overall survival and/or recurrence free survival. Patients belonging to the high-risk group were associated with poor outcomes, and the risk score was significant after adjusting for confounders. Validation of representative snoRNAs (SNORD46 and SNORD89) using qRT-PCR confirmed the observations from sequencing experiments. We also observed 64 snoRNAs harboring piwi-interacting RNAs and/or microRNAs that were predicted to target genes (mRNAs) involved in tumorigenesis. Our results demonstrate the potential of snoRNAs to serve (i) as novel prognostic markers for BC and (ii) as indirect regulators of gene expression.

## Introduction

Breast cancer (BC) is a complex polygenic disease [1] characterized by molecular and histological heterogeneity [2]. Although the diagnostic and prognostic factors related to BC outcomes are being increasingly refined, there remains a need to improve on the specificity and sensitivity of prognostic markers which may impact the quality of life for BC patients. Optimal management of BC is challenging due to the varied treatment response patterns exhibited by patients undergoing similar treatment regimens [3,4]. However, the available treatment modalities might be better applied if we could stratify treatment responders from non-responders, which may eventually help in improving survival and quality of life. Although estrogen, progesterone and human epidermal growth factor receptors are routinely used as prognostic markers, in addition to tumor and patient related factors, these indices remain as imperfect estimators for risk of recurrence and/or death [5]. Therefore, there is an ongoing search for better prognostic markers for BC.

With the discovery of new classes of small non-coding RNAs, their functions are ever expanding. Among the many small non-coding RNAs identified so far, microRNAs (miRNAs) are well established as global regulators of gene expression [6–10] that have also been studied comprehensively as biomarkers for various cancer types [11–16]. On the contrary, one of the lesser-studied classes of small non-coding RNAs is the group of small nucleolar RNAs (snoRNAs), which are approximately 60–300 nt in length [17]. snoRNAs often originate within the nucleolus of a cell and are mostly encoded within the intronic regions of protein-coding or non-protein coding genes such as long non-coding RNAs, or are independently transcribed from the intergenic regions [18]. snoRNAs are broadly classified into two groups: SNORAs, containing H/ACA box; and SNORDs, containing C/D box [19]. scaRNAs, or small Cajal body RNAs, can also be classified as snoRNAs [20]. snoRNAs are involved in ribosomal RNA (rRNA) maturation and biogenesis and also in modifications of other RNAs such as rRNAs, transfer RNAs (tRNAs) and small nuclear RNAs (snRNAs). Specifically, SNORAs are involved in pseudouridylation through their association with dyskerin protein and SNORDs, along with fibrillarin proteins, are involved in methylation. Nevertheless, not all snoRNAs have defined functions and are called “orphan snoRNAs” [20].

While the snoRNAs are largely recognized for performing housekeeping functions, emerging evidence suggests that dysregulation of snoRNAs occurs in various diseases. The first indication of the pathological importance of snoRNAs arose from the observation that a genetic locus containing snoRNAs was deleted in Prader Willi syndrome, a neurodevelopmental genetic disorder [21]. snoRNA deregulation has been observed in metabolic stress disorder [22] and in several cancer types including chronic lymphocytic leukemia [23], hepatocellular carcinoma [24], colorectal cancer [25] and endometrial cancer [26]. Their roles as diagnostic and prognostic biomarkers have been studied in colorectal cancer [25] and lung cancer [27,28]. Although reports by Dong et al [29] and Su et al [30] have implicated the importance of snoRNAs in breast carcinogenesis, a comprehensive understanding of snoRNAs as prognostic markers for BC is still lacking. snoRNAs are also beginning to be understood as indirect regulators of gene expression. snoRNAs may get processed to other smaller regulatory RNAs such as miRNAs and piwi-interacting RNAs (piRNAs), which are well known as post-transcriptional gene regulators [17,31,32].

We hypothesized that deregulation of snoRNAs contributes to inter-individual differences in BC trajectory and eventual outcomes. In this study, we investigated the potential of snoRNAs as prognostic markers for BC, focusing on overall survival (OS) and recurrence free survival (RFS). We have also explored the possible regulatory functions of snoRNAs. To the best of our knowledge, this is the first study to identify snoRNAs as potential independent prognostic markers for BC.

## Materials and Methods

### Ethics statement

Written informed consent was obtained from all the individuals who participated in this study. Local institutional research ethics committee (Health Research Ethics Board of Alberta: Cancer Committee) approved the study protocol.

### Breast tissue samples for the study

Breast tissue samples (control samples) were collected from 11 apparently healthy normal individuals who underwent reduction mammoplasty surgery and were flash frozen (FF) under 30 minutes of post-devitalization. The normal breast tissue specimens were obtained from Alberta Cancer Research Biobank (http://www.acrb.ca/). Samples from 104 pathologically confirmed invasive ductal carcinoma breast tumor tissues (cases) were obtained as formalin fixed paraffin embedded (FFPE) specimens from the same biobank. Detailed clinical characteristics of the study samples (collected between 1996 and 2008) have been documented in a previously described study [13]. Follow-up of the patients (median follow up = 8.02 years) indicated 61 recurrences and 46 deaths. All tumor tissue specimens had a tumor cellularity of at least 70%. We required at least eight samples in each group to identify snoRNAs with at least a two-fold difference, with a power of 80% and with α = 0.05 [13,33,34]. Earlier studies have demonstrated similar composition of snoRNAs from both FF and FFPE tissue specimens, suggesting that snoRNA expression may be comparable between FF and FFPE tissues [35].

### snoRNA profiling using small RNA sequencing

Details on RNA isolation and sequencing protocols are elaborated in our previous study [13,36]. The RNA isolation protocol involved DNAse I digestion step to remove potential genomic DNA contamination. Next generation sequencing (NGS) was performed at PlantBiosis Ltd (Lethbridge, Alberta, Canada; http://www.plantbiosis.com/). The data generated for the study was deposited in gene expression omnibus and the accession ID is GEO68085. Briefly, total RNA was isolated from cases and controls using TRIzol/Qiagen RNeasy kit and RecoverAll Total Nucleic Acid Isolation kit (Life Technologies), respectively. Small RNA libraries were generated using TruSeq small RNA library construction protocol with no modifications. The protocol aims to select and amplify small RNAs, between 15–40 nt in length. The libraries were subjected to small RNA sequencing using Illumina Genome Analyzer IIx with 36 cycles single end protocol. One tumor sample could not be processed further due to quality reasons, leaving 103 tumor samples and 11 normal samples for further analysis. Base calling and demultiplexing was performed using CASAVA 1.8.2, followed by adapter trimming using CutAdapt software (https://cutadapt.readthedocs.org/). Bowtie [37] was used for aligning the trimmed reads to hg19 genomic assembly (downloaded from Illumina iGenome repository). The generated.sam files were converted to.bam files, which were used for subsequent analysis using Partek Genomics Suite 6.6 (PGS, Partek Genomics Suite software, version 6.6 beta, Partek Inc., St. Louis, MO, USA). snoRNAs were annotated using Ensembl database [38].

### Statistical analysis to identify potential prognostic markers

Two independent and proven approaches in a biomarker study are the Case-control (CC) and the Case-only (CO) approaches. While it is common to see either of the two approaches in literature [16,39–42], we have adopted both methods in our study to identify the most suitable approach to conduct a biomarker study. The two approaches were employed to select the list of snoRNAs for survival analysis. In the CC approach, both normal (controls) and tumor (cases) samples were analyzed, whereas in the CO approach, only the tumor samples were analyzed. For both methods, the datasets were normalized using reads per kilobase per million method (RPKM) [43] and were adjusted for batch effects using one-way ANOVA model. Profiled snoRNAs with at least one read count in any one of the samples were considered. The datasets were further filtered for read counts: only snoRNAs with at least 10 read counts in 90% of the samples (normal and tumor inclusive for CC and tumor for CO) were retained for downstream analysis. In the CC approach, only differentially expressed (DE) snoRNAs with a stringent threshold of a fold change (FC) > 2.0 and a false discovery rate (FDR) ≤ 0.05 were considered for survival analysis. However, in the CO approach, all the snoRNAs retained after filtering were considered for survival analysis, as described earlier [13]. We performed Univariate Cox proportional hazards regression analysis for overall survival (OS) and for recurrence free survival (RFS), followed by permutation test (n = 10,000), considering the snoRNAs (DE snoRNAs from CC and the filtered snoRNAs from the CO approaches) as continuous variables. snoRNAs with permutation p-values ≤ 0.1 were used for constructing risk scores for all samples. Risk scores were constructed using the formula:

${Risk\;Score}_{i}=\sum_{j=1}^{12}\beta_{j}*{snoRNA}_{ij}$; where *snoRNA_ij_* is the individual risk score for snoRNA j on sample i, and *β_j_* is the parameter estimate obtained from the univariate analysis for snoRNA j [16]. Further, the risk-scores obtained were dichotomized into low-risk and high-risk groups based on the optimal cut-off point estimated using receiver operating characteristics curve (ROC). The constructed risk scores were considered as dichotomous variables and a multivariate Cox proportional hazards regression analysis was performed along with other potential confounders: age at diagnosis (continuous variable), tumor stage (I, II vs. III, IV), tumor grade (high vs. low) and triple negative breast cancer status (TNBC vs. Luminal). The final multivariate model included the variables which were significant at p<0.05 and it was the same for OS and RFS outcome. Kaplan-Meier plots along with log-rank test were used for assessing the median survival function and for comparing the survival distributions between low-risk and high-risk groups, respectively. All the analyses except survival analysis were conducted using Partek Genomics Suite v 6.6. Survival analysis tests were performed in SAS (SAS institute Inc., Cary, NC) version 9.3, and statistical significance was defined as p < 0.05. Permutation test was performed in R statistical program using the “glmperm” package and p ≤0.1 was considered statistically significant.

### Technical validation of snoRNA expression using qRT-PCR

The expression of two representative snoRNAs showing prognostic significance (SNORD46 and SNORD89) were validated with total RNA isolated from a subset of samples used for sequencing. Amongst the prognostically significant snoRNAs, SNORD46 and SNORD 89 showed the highest fold changes and were therefore considered for cross platform validation. Real time quantitative reverse transcription polymerase chain reaction (qRT-PCR) was performed using an iScript Select cDNA Synthesis Kit (Bio-Rad) and a SsoFast EvaGreen Supermix (Bio-Rad) according to manufacturers’ instructions. Reverse transcription of total RNA was performed using random primers. Primers for PCR amplification of SNORD46 and SNORD89, designed using Primer3 software, were as follows: SNORD46-F: 5’-AAT CCT TAG GCG TGG TTG TG-3’, SNORD46-R: 5’-ATG ACA AGT CCT TGC ATT GG-3’; and SNORD89-F: 5’-GAC AAG AAA AGG CCG AAT TG-3’, SNORD89-R: 5’-CAT GGA GAG CAA ACT GCT GA-3’. RNU6-2 served as loading control and the primer sequences were RNU6-2-F: 5’-CGC TTC GGC AGC ACA TAT AC-3’, RNU6-2-R: 5’-AGG GGC CAT GCT AAT CTT CT-3’. All assays were done in triplicates, data was analyzed using the 2^-ΔΔCt^ method [44], and results are shown as fold induction of snoRNAs.

### Gene (mRNA) expression analysis

We downloaded the breast tissue gene (mRNA) expression dataset (GEO accession ID: GSE22820) which was originally generated in-house; briefly 141 breast tumor samples and 10 normal breast tissues obtained from reduction mammoplasty [13,45] were profiled using Agilent platform. Partek Genomics Suite v6.6 served as a tool for gene expression analysis. The raw intensity files were quantile normalized and log2 transformed. Differentially expressed (DE) genes were identified as those exhibiting FC > 2.0 and FDR ≤ 0.05 using one-way ANOVA.

Targets for piRNAs embedded within snoRNAs were identified using miRanda algorithm v 3.3a. Fasta sequences of the 3’ untranslated region (UTR) of all the DE genes identified from the in-house BC gene expression dataset were downloaded from Ensembl database (GRCh37) [38] and fasta sequences of the 11 piRNAs were downloaded from piRNA Bank (hg 19) [46]. Since piRNAs and mRNAs are known to exhibit reciprocal relationships (i.e., if a piRNA is up-regulated, the gene target is down-regulated and vice-versa) [36,47], targets for down-regulated piRNAs (obtained from our previous study) [36] were interrogated from the list of up-regulated genes using miRanda. Likewise, targets for up-regulated piRNAs were interrogated from the list of down-regulated genes. Only genes from piRNA-mRNA pairs with alignment score ≥ 170 and energy threshold ≤ -20 kcal/mol [36,47] were considered for gene ontology classification.

## Results

### 40 snoRNAs are differentially expressed in BC

As described in our previous study [13], 10,016,964 and 164,237,348 reads were obtained from normal and tumor tissues, respectively. Of these, 5,060,588 and 97,204,377 reads were retained after adapter trimming in normal and tumor tissues, respectively. Among the reads that aligned to the human genome (4,255,616 in normal and 84,240,355 in tumor), 1,610,928 reads (163,459 in normal and 1,447,469 in tumor) belonged to snoRNAs, and annotated to 768 snoRNAs. Since full length snoRNAs are > 60nt in length, it is unlikely that the sequencing protocol used in this study would have captured these snoRNAs. Therefore the snoRNAs that we have profiled are likely to be the fragments, whose reads mapped to the 5’ or 3’ ends of full length snoRNAs. Read distribution of representative snoRNAs (from the 13 prognostically significant snoRNAs identified in this study) are illustrated in S1 Fig. The read distributions confirm that the identified snoRNA fragments are not unique to FFPE tissues, as the FF normal reduction mammoplasty tissues also exhibited these characteristics, negating the view that storage of the samples under different conditions would have generated the fragments. However, the reason for the generation of endogenous snoRNA fragments is not clear. Four samples were classified as outliers in principal component analysis, leaving data from 99 tumor samples for downstream analysis.

**Fig 1:**
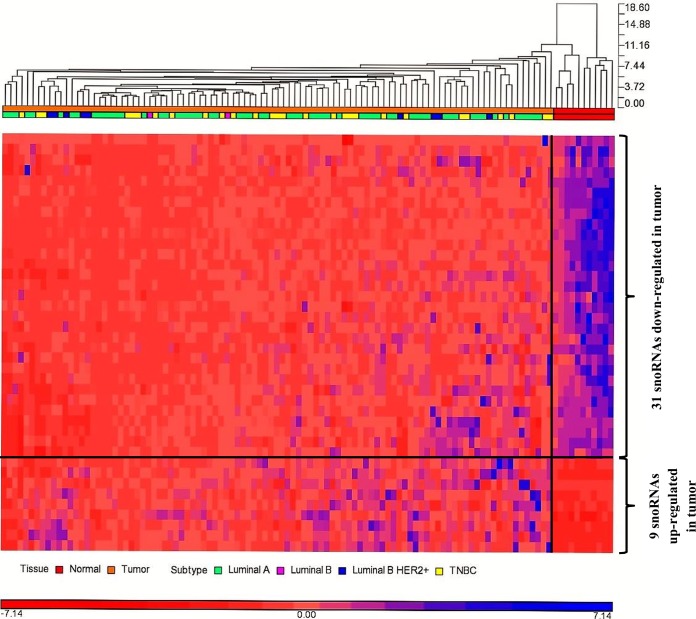
Hierarchical clustering of differentially expressed snoRNAs. The 40 differentially expressed snoRNAs were subjected to unsupervised hierarchical clustering with average linkage and Euclidean as distance measure. The tumor samples (orange horizontal bars) were clearly separated from the normal samples (red horizontal bars).

There were 88 snoRNAs retained after filtering for read counts in the CC approach. The dataset was RPKM normalized and corrected for batch effects (S2 Fig). The raw counts of the 768 snoRNAs and the batch adjusted normalized counts of all snoRNAs and filtered snoRNAs are provided in S1A–S1C Table. Among the 88 filtered snoRNAs, 40 snoRNAs were DE (FC > 2.0, FDR ≤ 0.05, S2 Table); 77.5% (n = 31) of which were down-regulated in tumor (Fig 1).

Further, to investigate if snoRNAs are stable in FFPE tissues over years, we chose samples that were collected in 1996 and 2008 (the oldest and the most recently collected samples) and ran a Pearson’s correlation test on the raw and normalized counts of filtered snoRNAs (n = 88). We obtained strong correlations for both raw and normalized data, with corresponding Pearson correlation coefficients of r = 0.801 and r = 0.913, respectively, indicating the stability of the snoRNAs from FFPE tissues profiled in this study (S3 Fig). This observation from our dataset is supported by findings from Hall et al., who have identified snoRNAs as one of the stable molecules from FFPE tissue samples [48].

### Thirteen snoRNAs identified with prognostic relevance for breast cancer

For the CC approach, 40 DE snoRNAs were subjected to survival analysis, whereas, for the CO approach, 95 snoRNAs, which were retained after filtering for read counts (from a total of 763 snoRNAs), were subjected to survival analysis. The raw counts of all 763 snoRNAs and the batch adjusted normalized counts of 763 and 95 filtered snoRNAs, obtained from the CO approach are provided in S1D–S1F Table. The 40 DE snoRNAs and the 95 snoRNAs from the CO approach were first analyzed as continuous variables and were tested for their association with OS and RFS, followed by permutation test for univariate cox model. For OS, 12 snoRNAs were found to have permutation p-values ≤ 0.1 in the CO approach, which also included the five significant snoRNAs identified from the CC approach (S3 Table). Similarly, for RFS, 10 snoRNAs were identified from the CO approach that included four snoRNAs from the CC approach (S3 Table). Overall, we identified 13 non-redundant snoRNAs associated with prognosis.

**Fig 2:**
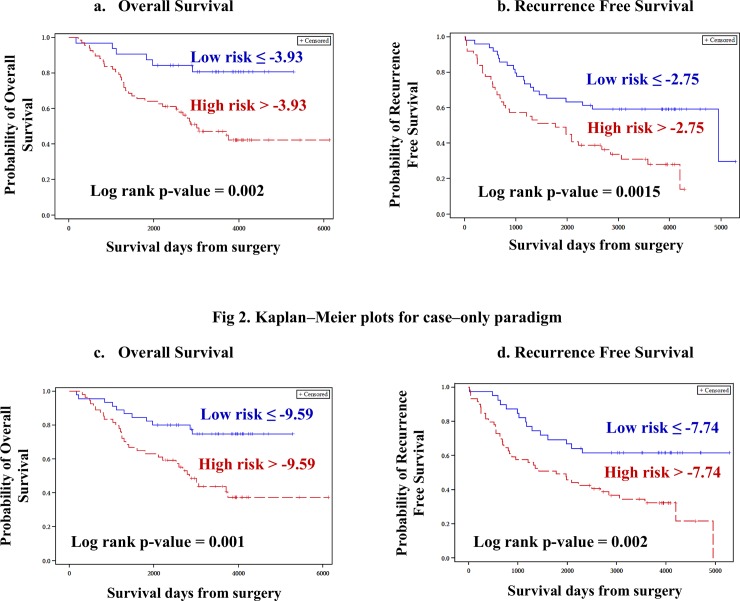
Kaplan–Meier plots for case–control approach. Kaplan-Meier plots for risk scores were constructed to determine survival differences between low–risk and high–risk groups. Significant survival differences existed between the two risk groups, as indicated by the log–rank p–values. (A) OS for CC approach. (B) RFS for CC approach. (C) OS for CO approach and (D) RFS for CO approach. In all these approaches, patients belonging to high–risk group showed poor OS and RFS.

For both OS and RFS, risk scores were computed individually for the CC and CO approaches for every sample. For the CC approach, -3.93 and -2.75 were estimated to be the optimal cut-off points for OS and RFS, respectively, separating BC patients into low-risk and high-risk groups. Likewise, for the CO approach, -9.59 and -7.74 were estimated as optimal cut-off points for OS and RFS, respectively for patient dichotomization into risk groups. Risk scores were considered as dichotomous variables and were entered into univariate and multivariate Cox proportional hazards regression models. In both CC (Table 1A, Fig 2A and Fig 2B) and CO (Table 1B, Fig 2C and Fig 2D) approaches, patients belonging to the high-risk groups were associated with shorter OS and RFS and risk scores emerged significant after adjusting for potential confounders.

**Table 1:** Univariate and multivariate results.

**A. Case–control approach**								
**Parameter**	**Overall Survival**				**Recurrence Free Survival**			
	**Univariate**		**Multivariate**		**Univariate**		**Multivariate**	
	**HR(95% CI)**	**P–value**	**HR (95% CI)**	**P–value**	**HR(95% CI)**	**P–value**	**HR(95% CI)**	**P–value**
**Risk score**	3.59(1.51–8.54)	0.004	3.24(1.35–7.77)	0.008	2.38(1.37–4.14)	0.002	2.17(1.22–3.84)	0.008
**Tumor stage**	0.39(0.2–0.78)	0.007			0.42(0.22–0.78)	0.007		
**Tumor grade**	2.15(1.06–4.39)	0.035	2.19(1.07–4.52)	0.033	1.61(0.91–2.86)	0.1		
**Age at diagnosis**	1.06(1.02–1.09)	0.001	1.05(1.02–1.09)	0.003	1.02(0.99–1.05)	0.2		
**TNBC status**	0.93(0.46–1.89)	0.83			0.76(0.4–1.45)	0.41		
**B. Case–only approach**								
**Parameter**	**Overall Survival**				**Recurrence Free Survival**			
	**Univariate**		**Multivariate**		**Univariate**		**Multivariate**	
	**HR(95% CI)**	**P–value**	**HR(95% CI)**	**P–value**	**HR(95% CI)**	**P–value**	**HR(95% CI)**	**P–value**
**Risk score**	2.95(1.48–5.88)	0.002	2.75(1.37–5.52)	0.005	2.44(1.35–4.43)	0.003	2.42(1.33–4.42)	0.004
**Tumor stage**	0.39(0.2–0.78)	0.007			0.42(0.22–0.78)	0.007		
**Tumor grade**	2.15(1.06–4.39)	0.035	2.15(1.04–4.42)	0.038	1.61(0.91–2.86)	0.1		
**Age at diagnosis**	1.06(1.02–1.09)	0.001	1.06(1.02–1.09)	0.002	1.02(0.99–1.05)	0.2		
**TNBC status**	0.93(0.46–1.89)	0.83			0.76(0.4–1.45)	0.41		

HR = Hazard ratio; CI = Confidence interval; TNBC = Triple negative breast cancer. (A) The risk scores computed for the CC (one for OS and one for RFS) and (B) the risk scores computed for CO (one for OS and one for RFS) approaches were significant in the multivariate analysis (p < 0.05) after adjusting for potential confounders. In both approaches, patients with risk scores more than the estimated optimal cut–off points were associated with poor prognosis (HR > 1).

### Concordance of findings between NGS and qRT-PCR

**Fig 3:**
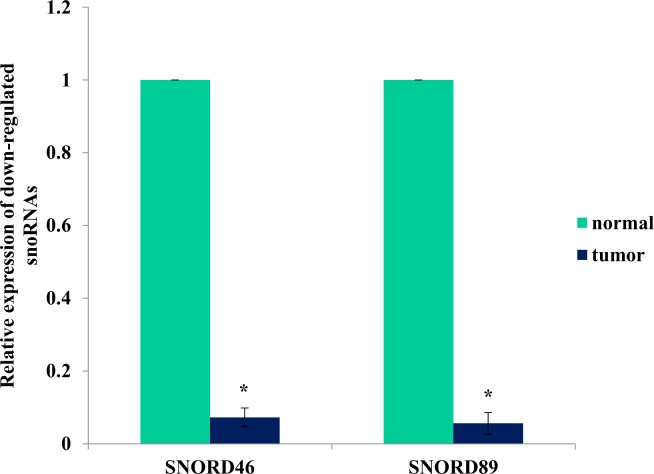
qRT-PCR confirmation of snoRNA expression. SNORD46 and SNORD89 were confirmed to be down–regulated in tumor, relative to normal samples using qRT-PCR platform. The Ct values obtained for snoRNAs were normalized to Ct values obtained for RNU6. * indicates statistical significance p<0.05.

In NGS analysis, SNORD46 and SNORD89 were found to be down-regulated in tumors, relative to normal samples, with fold changes of -7.38 and -4.07, respectively. When analyzed using qRT-PCR, these two snoRNAs showed the same direction of expression—i.e., both RNAs were down-regulated in tumor tissues, relative to normal samples (p < 0.05), confirming the findings from NGS (Fig 3). SNORD 46 and SNORD89 were found to be embedded within the intronic regions of RPS8 and RNF149 genes, respectively. Since we have used random primers (and not oligo-dT primers) for reverse transcription, the primary source of the transcript needed to be ascertained. Therefore to ensure that the PCR products are not from the host transcripts (pre-mRNA), we interrogated the expression of RPS8 and RNF149 in the breast tissue gene (mRNA) expression dataset. We found that RPS8 was up-regulated in tumor tissues (FC = 1.4). This is in contrast with the expression of SNORD46, which was found to be down-regulated. On the other hand, we did not observe any expression changes in the RNF149 gene (when SNORD89 showed down-regulation in tumor tissues relative to normal tissues). The discordant expression patterns rule out the possibility that random primers may have contributed to the cDNA representing the host (pre-mRNA) transcript.

### Insights into the regulatory functions of snoRNAs

Previous studies have reported that snoRNA genes are often found within the intronic regions of protein-coding and non-protein coding genes, (snoRNA host genes) [18]. We also observed that out of 768 snoRNAs that were profiled in breast tissues (including normal and tumor tissues), 449 (i.e., > 50%) snoRNAs mapped to the intronic regions of protein coding genes (S4A Table). It has also been demonstrated that snoRNAs can act as a source for other regulatory small non-coding RNAs, such as miRNAs [17,31,32] and piRNAs [49], implying a novel function and/or biological relevance for snoRNAs in gene regulation. In this study, we overlapped the genomic coordinates of all 768 snoRNAs with those of mature miRNAs obtained from miRBase v 20. We observed that six snoRNAs harbored eight mature miRNAs. Further, we compared the direction of fold change between these miRNA-snoRNA pairs [13] and observed that five were expressed in the same direction in tumor tissues, relative to normal tissues (S4B Table), hinting at the possibility that these miRNA-snoRNA pairs may be co–regulated. We also extended this comparison to piRNAs and observed that 58 snoRNAs harbored piRNAs (S4C Table). Of these, 35 piRNA-snoRNA pairs were expressed in the same direction in tumors, relative to normal tissues—i.e. if the piRNA was up-regulated in tumor tissues, its corresponding host snoRNA was also up-regulated in tumor tissues (S4C Table). Additionally, from among the 35 pairs, 11 piRNAs were DE with FC > 2.0 and FDR ≤ 0.05 (Table 2) [36], of which six were down-regulated and five were up-regulated in tumor tissues, relative to normal tissues. We identified gene targets regulated by these piRNAs. Analysis of the breast tissue gene expression dataset (refer to methods) yielded 628 up-regulated genes and 2241 down-regulated genes. Targets for the six down-regulated and five up-regulated piRNAs were interrogated using the 628 up-regulated and 2241 down-regulated genes, respectively. piRNA-mRNA targets with the specified criteria of alignment score and energy threshold score are summarized in Table 2. Gene ontology classifications of the genes identified as targets for piRNAs are summarized in S5 Table. We did not identify the gene targets for miRNAs because only one miRNA had a fold change of > 2.0 (predefined cut-off).

**Table 2:** snoRNA-piRNA pairs with same direction of expression, fold change > 2.0 and FDR ≤ 0.05.

Host gene	snoRNA ID(Fold change)	Target RNA for snoRNA	piRNA embedded within snoRNA (Fold change)	mRNA targets for the embedded piRNAs
NOP56	SNORD110-201(-24.22)	18S rRNA U1288	hsa_piR_019676(-8.01)	DGKH,CLEC5A,ADAMDEC1,HOXC13,LRRC15,IQCH,WDR62
SNHG24	SNORD114-23-201(-4.39)	unknown	hsa_piR_019102(-6.43)	BPNT1,CASC5,KIF26B,PRAME,TLL2,ZC3H12D
SNX5	SNORD17-201(-2.12)	28S rRNA U3797	hsa_piR_017033(-2.17)	MAGEA4,PLGLB2,TNFSF4,FAM83D,CGA,FOSL1,GAS2L3,BRIP1,NCAPG,PLGLB2
HSPA9	SNORD63-201(-3.80)	28S rRNA A4541	hsa_piR_000586(-3.83)	None
AP1G1	SNORD71-201(-2.09)	5.8S rRNA U14	hsa_piR_002158(-2.78)	TPM3,DQX1
DDX39B, ATP6V1G2-DDX39B	SNORD84-201(-2.24)	unknown	hsa_piR_001078(-4.79)	GRM4,CENPI,CHRNA1,GPR26
TPT1	SNORA31-001(1.58)	18S rRNA U218 and 28S rRNA U3713	hsa_piR_017184(9.17)	TMEM47,TRPM3,ZNF462
PRRC2A	SNORA38-201(14.44)	unknown	hsa_piR_004531(54.1)	SLC34A1,SLC6A2,SEC31B,TFAP2C,TTC23,TXNIP,XPNPEP3,CNTN2
MRPL3	SNORA58-001(8.65)	28S rRNA U3823	hsa_piR_020466(4.06)	SLC27A1
SNHG16	SNORD1B-201(5.08)	28S rRNA G4362	hsa_piR_018780(18.45)	SMAD2,TNRC6B,TRIM9,UTRN,USP6,ANGPTL1,ARHGAP6,ALB,BCHE,CNTNAP3
CCAR1	SNORD98-201(2.79)	18S rRNA G867	hsa_piR_000045(4.85)	SFRP1,RSPO3,SPARCL1,WSCD1,ADRA2A,AVPR1A,ASPH,BCL6,CCDC25

The host genes indicate the genes within which the snoRNAs are embedded. Since snoRNAs are involved in the modification of other RNAs, we have also indicated the target RNAs of the 11 snoRNAs. Of the 35 piRNAs found to be harbored within snoRNAs, 11 piRNAs were observed to be DE with a fold change > 2.0 and FDR ≤ 0.05. Since piRNAs are involved in gene regulation, the target mRNAs are listed corresponding to its piRNA.

## Discussion

In this study, we identified 13 snoRNAs as potential novel prognostic markers for BC. Twelve snoRNAs were found to be associated with OS and ten snoRNAs were found to be associated with RFS, among which nine were common between OS and RFS for BC. We also explored their potential roles in gene regulation. snoRNAs are well known to be involved in post-transcriptional modification of other regulatory non-coding RNAs. Alternative roles of snoRNAs such as their association with various clinical factors or their involvement in gene regulation are also emerging [23–26,31,32,49].

Our study design included two approaches (CC and CO) to identify the appropriate method for discovering prognostic markers. While the CC approach tests only the DE snoRNAs for association with outcomes [12,39], the CO approach is unbiased and interrogates all the snoRNAs retained after filtering, and is independent of the control tissues used [14,16]. Composite risk scores were calculated for the following reasons: (i) individual markers are not adequate to capture the complex interactions involved in conferring phenotypes and (ii) inclusion of all snoRNAs significant in the univariate analysis may contribute to data overfitting. The constructed risk scores were identified as potential independent prognostic factors for BC. Overall, in the CC approach, we identified a total of six non-redundant snoRNAs associated with disease outcomes (OS and RFS included). As expected, we identified a higher number of snoRNA markers (n = 13) from the CO approach, which also included signatures identified from the CC approach. The same pattern of identifying higher number of markers in the CO approach (including those identified from the CC approach) was observed when we interrogated this dataset for miRNAs and piRNAs as prognostic markers [13,36]. Our results highlight the importance of considering the CO approach for a biomarker study.

To the best of our knowledge, this is the first study to report snoRNAs as prognostic markers for BC. In fact, none of the prognostic snoRNAs identified in this study have been reported in any of the other cancer types analysed thus far. These potentially novel biomarkers need to be validated in independent studies to ascertain their role in BC prognostication. However, at this time, it is not certain if the 13 prognostic snoRNAs are specific to BC or if they share prognostic relevance in other cancer types. It is possible that with more genome-wide studies focusing on understanding the clinical relevance of snoRNAs, we may be able to identify these snoRNAs in other cancer types. It would also be interesting to see if the identified snoRNAs show any subtype or tumor stage or grade specificity. In this pilot study conducted using 104 tumors; 62 samples belonged to Luminal A subtype (26 deaths and 37 recurrences) and 30 belonged to TNBC subtype (11 deaths and 13 recurrences). Given the current sample size and the number of events, it was not feasible to conduct further finer analysis based on stratified subtypes of BC.

**Fig 4:**
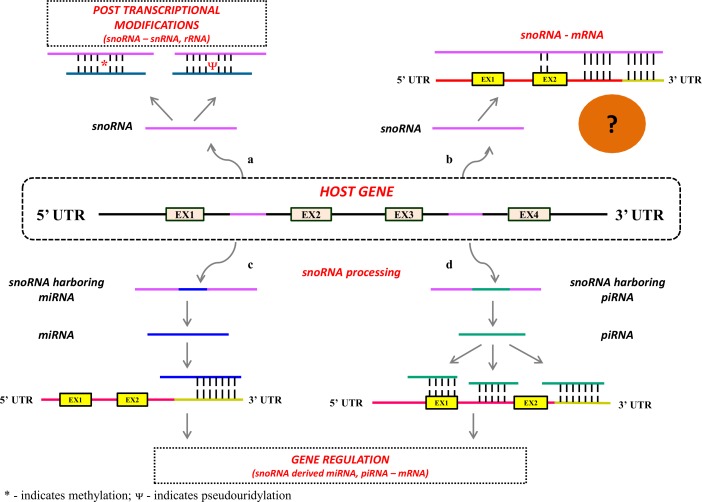
Complex interplay of snoRNAs with other RNAs. snoRNAs are involved in diverse biological functions. They arise from the intronic regions of protein coding / non-protein coding genes (host genes). EX represents exons. Black lines indicate intronic regions and purple lines within intronic regions indicate the coding regions for snoRNAs. The canonical function of snoRNAs is its role in post-transcriptional modifications of snRNAs and rRNAs, which are involved in splicing mechanism and protein translation, respectively (a). One of the emerging roles of snoRNAs is its involvement in gene regulation. snoRNAs may act as a source for other small RNAs such as miRNAs (b, indicated in deep blue) and piRNAs (c, indicated in green). miRNAs and piRNAs are considered as master regulators of gene expression that may bind to the untranslated regions (3’ UTR or 5’ UTR), exons or introns and may promote either mRNA degradation or translation inhibition; implying the indirect role of snoRNAs in gene regulation. (d). The other unknown function of snoRNAs is its direct interaction with mRNAs through complementary base pairing. To-date, the direct interaction of snoRNAs with mRNAs has not been studied; however, this interaction might be a possibility based on the demonstrated subsets of snoRNAs embedding piRNAs and miRNAs, and their interactions with mRNAs through base pair complementarities; further research into this field may enhance our understanding on the direct role of snoRNAs in gene regulation.

We understand that a complex interplay exists between different classes of RNAs for normal developmental processes and for maintaining homeostasis. For instance, snoRNAs are known to be embedded within the intronic regions of protein-coding or non-protein coding genes. The well-studied function of snoRNAs includes participation in post-transcriptional modifications of other RNAs such as ribosomal RNAs (involved in protein translation), small nuclear RNAs (involved in splicing mechanisms) and transfer RNAs (involved in protein translation). However, understanding of snoRNAs is slowly expanding towards gene regulation. snoRNAs have not been found to interact directly with mRNAs causing translational repression or mRNA degradation, similar to miRNAs. An alternative mechanism has been suggested, wherein the snoRNAs may get processed to form other regulatory RNAs such as miRNAs and piRNAs, well established regulators of gene expression. Fig 4 and Table 2 illustrate the complex interplay of these RNAs. In our dataset, we found 450 snoRNAs to be embedded within the intronic regions of protein-coding genes (S4A Table), and 8 miRNAs (S4B Table) and 58 piRNAs (S4C Table) to be present within the genomic boundaries of snoRNAs. We also observed that the 11 snoRNA-piRNA pairs reported (Table 2) showed the same direction of alteration in tumor tissues–i.e., if the snoRNA was down-regulated in the tumor tissues, its corresponding piRNA was also down-regulated. It could be speculated that some of the snoRNAs and piRNAs may be co-regulated and may share a common promoter. However, the processing of these piRNAs/miRNAs from the snoRNAs needs to be ascertained, and further experiments are needed to understand their co-regulation, if any.

Since these piRNAs originated from within the snoRNAs, the snoRNAs also shared certain degree of complementarity with the mRNAs (data not shown). It is not known if this degree of complementarity implies a direct interaction between snoRNAs and mRNAs and thus contributes to direct gene regulation. snoRNAs are larger in size (60–300 nt) than other regulatory small RNAs (miRNAs and piRNAs, 18–30nt). Therefore, the immediate challenge is to determine if canonical seed sequence motifs exist for snoRNAs to mediate gene silencing effects. However, at this point of time, we know that ectopic expressions of snoRNAs in a cell line or animal model could contribute to various cancer characteristics such as cell proliferation, invasion and migration [25,50,51]. Interestingly, high expression of ACA11 was also found to contribute to increased resistance to chemotherapy in multiple myeloma [50], suggesting that snoRNAs may be important players for tumorigenesis. The targets identified for the 11 piRNAs (identified in our study) showed relevance in important tumorigenic pathways such as cell proliferation, cell adhesion and apoptosis (S5 Table). Functional validation studies are thus warranted to confirm if these piRNAs interact directly with their corresponding targets to promote gene silencing.

Overall, we profiled 768 snoRNAs from breast tissues and identified 40 snoRNAs as differentially expressed. However, the DE results should be interpreted with caution as we used normal samples preserved as FF tissues and tumor samples preserved as FFPE tissues. Therefore it is possible that the observed differences in snoRNA expression may have also arisen because of different tissue preservation techniques, as indicated by Martens-Uzunova et al [52]. Given the sequencing protocol adopted in this study (36 cycles single end protocol) with read lengths ranging between 17 and 27 nucleotides, it is highly likely that the 768 snoRNAs may not represent the entire snoRNAome. We performed size fractionation to include RNAs with a size range of 20-30nt and since full-length snoRNAs have a minimum length of 60 nucleotides, the identified snoRNAs may actually be fragments of snoRNAs. However, at this point of time, it is not clear if these fragments are products of snoRNA processing or if these are representative of full length snoRNAs and therefore we referred to these identified sequences as merely snoRNAs. Reading longer transcripts with higher number of sequencing cycles may help identify additional snoRNAs and to ascertain the origins of the profiled fragments. Despite these challenges, we have attempted a genome-wide profiling of snoRNAs and have demonstrated their potential as novel players for BC prognostication.

## Conclusions

In this study, we determined two aspects of snoRNAs: (i) their importance as prognostic markers for BC and (ii) their possible roles in gene regulation. We report 13 (non-redundant) novel promising prognostic markers for BC: 12 for OS and 10 for RFS. The contribution of snoRNAs to tumorigenesis is manifested through (i) their primary action in post-transcriptional modifications of other RNAs, and (ii) their processing to generate small RNAs that are directly involved in gene regulation. While the first contribution of snoRNAs is well established, their role in gene regulation is only just emerging. Insights into these aspects could open up new avenues for the development of snoRNAs for diagnostic and therapeutic purposes.

## Supporting Information

S1 Fig Read distribution of prognostically significant snoRNAs. snoRNAs captured in this study potentially reflect multiple fragments that map to 3’or 5’ends of snoRNAs, as shown from the read distribution of representative snoRNAs. Data represented are from the 13 prognostically significant snoRNAs, from both FFPE tissues and FF normal breast tissues from reduction mammoplasty.
(TIFF)

S2 Fig Detection of batch effects. The raw counts of all 768 snoRNAs were RPKM normalized and corrected for batch effects. S2A Fig represents the data before batch effects correction (Mean F ratio of batch = 19.73) and S2B Fig represents the data after batch effects correction (Mean F ratio of batch = 0). The factor ‘tissue’ represents biological variation arising from normal and tumor tissues; hence was not appropriate to correct for.
(TIF)

S3 Fig Stability of snoRNAs in FFPE samples. Scatter plots of 88 snoRNAs detected from a 16 year old sample (collected in 1996) and a 4 year old sample (collected in 2008). Correlation coefficients ≥ 0.8 from raw counts (a) and > 0.9 from batch adjusted normalized counts (b) indicate that the snoRNAs are stable in FFPE samples.
(TIF)

S1 Table Raw and normalized counts of snoRNAs. The sequenced and aligned data files (.bam files) were analyzed using PGS. The raw files were normalized using RPKM method which was adjusted for batch effects using ANOVA model. snoRNAs were further filtered for read counts: only snoRNAs with ≥ 10 read counts in at least 90% of the samples were retained for further analysis. Raw and normalized counts (for all the snoRNAs and for the filtered snoRNAs) obtained from the CC approach are summarized in S1A–S1C Tables and those obtained from the CO approach are summarized in S1D–S1F Tables.
(XLSX)

S2 Table List of 40 differentially expressed snoRNAs. snoRNAs filtered for read counts in the CC approach were subjected to one-way ANOVA test to identify differentially expressed snoRNAs with fold change > 2.0 and FDR cut off ≤ 0.05. Forty snoRNAs were differentially expressed; 9 showed up-regulation and 31 showed down-regulation in tumors, relative to normal tissues.
(PDF)

S3 Table List of snoRNAs with prognostic relevance for breast cancer. In the CO approach, twelve and ten snoRNAs were identified for OS and RFS, respectively with permutation p-value ≤ 0.1. The snoRNAs identified in the CO approach encompassed all the snoRNAs identified in the CC approach for both OS (n = 5) and RFS (n = 4) and are highlighted in red.
(PDF)

S4 Table List of snoRNAs embedded within protein-coding genes and snoRNAs harboring miRNAs and piRNAs. snoRNAs are known to arise from the intronic regions of protein-coding and non-protein-coding genes. In this study, we observed that of the 768 snoRNAs profiled from breast tissues, 449 snoRNAs (i.e., > 50%) mapped to the intronic regions of protein-coding genes (S4A Table). S4B and S4C Table represent snoRNAs harboring miRNAs and piRNAs, respectively.
(XLSX)

S5 Table Gene ontology terms associated with genes targeted by piRNAs embedded within snoRNAs. (PDF)
