# Supplementary figures and images for: Profiling of Small Nucleolar RNAs by Next Generation Sequencing: Potential New Players for Breast Cancer Prognosis
Source: PLoS One. 2016 Sep 15;11(9):e0162622. doi: 10.1371/journal.pone.0162622 (PMC5025248; doi:10.1371/journal.pone.0162622)

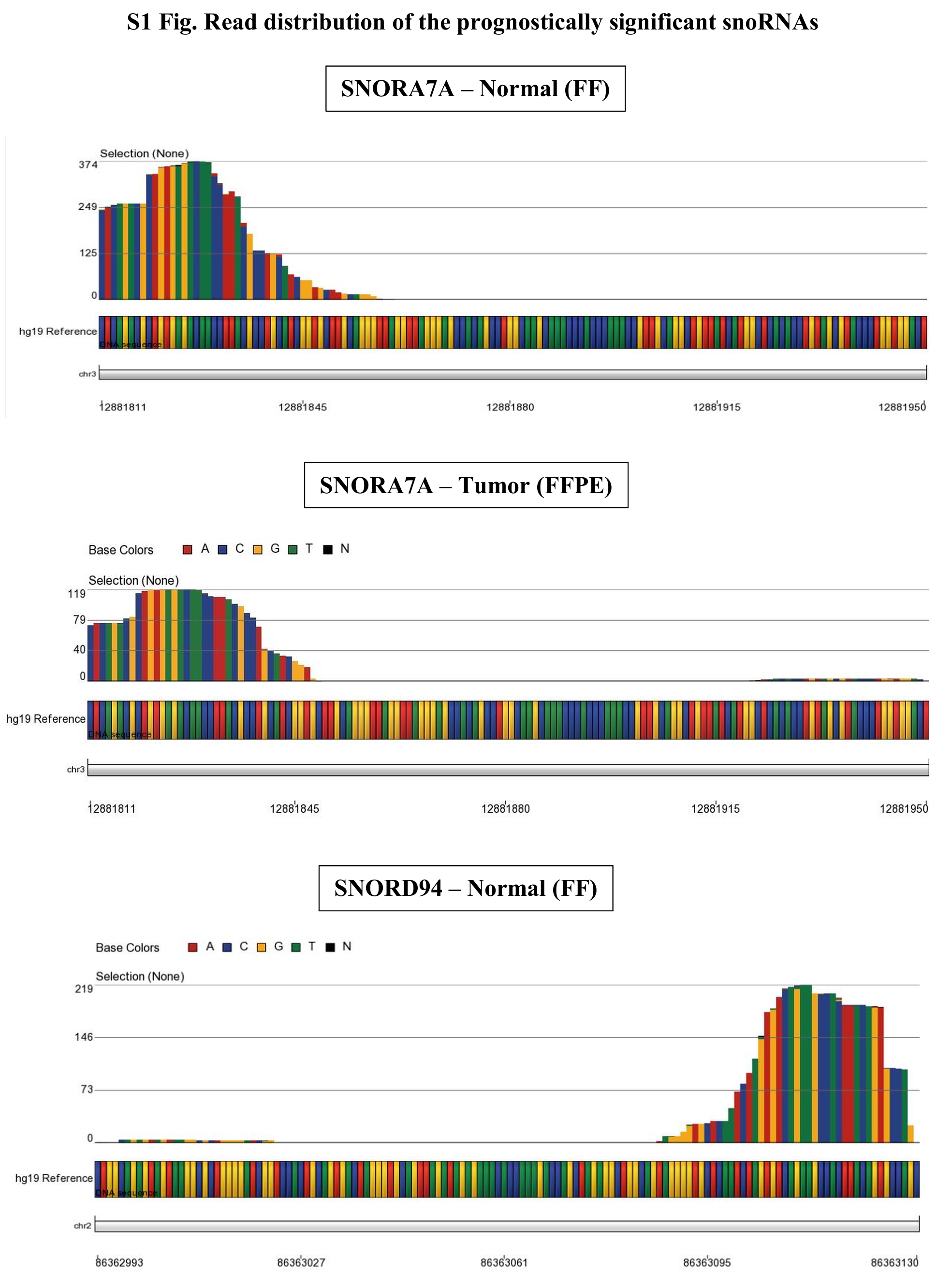

Supplement: S1 Fig — snoRNAs captured in this study potentially reflect multiple fragments that map to 3’or 5’ends of snoRNAs, as shown from the read distribution of representative snoRNAs. Data represented are from the 13 prognostically significant snoRNAs, from both FFPE tissues and FF normal breast tissues from reduction mammoplasty. (TIFF) [file pone.0162622.s001.tiff]

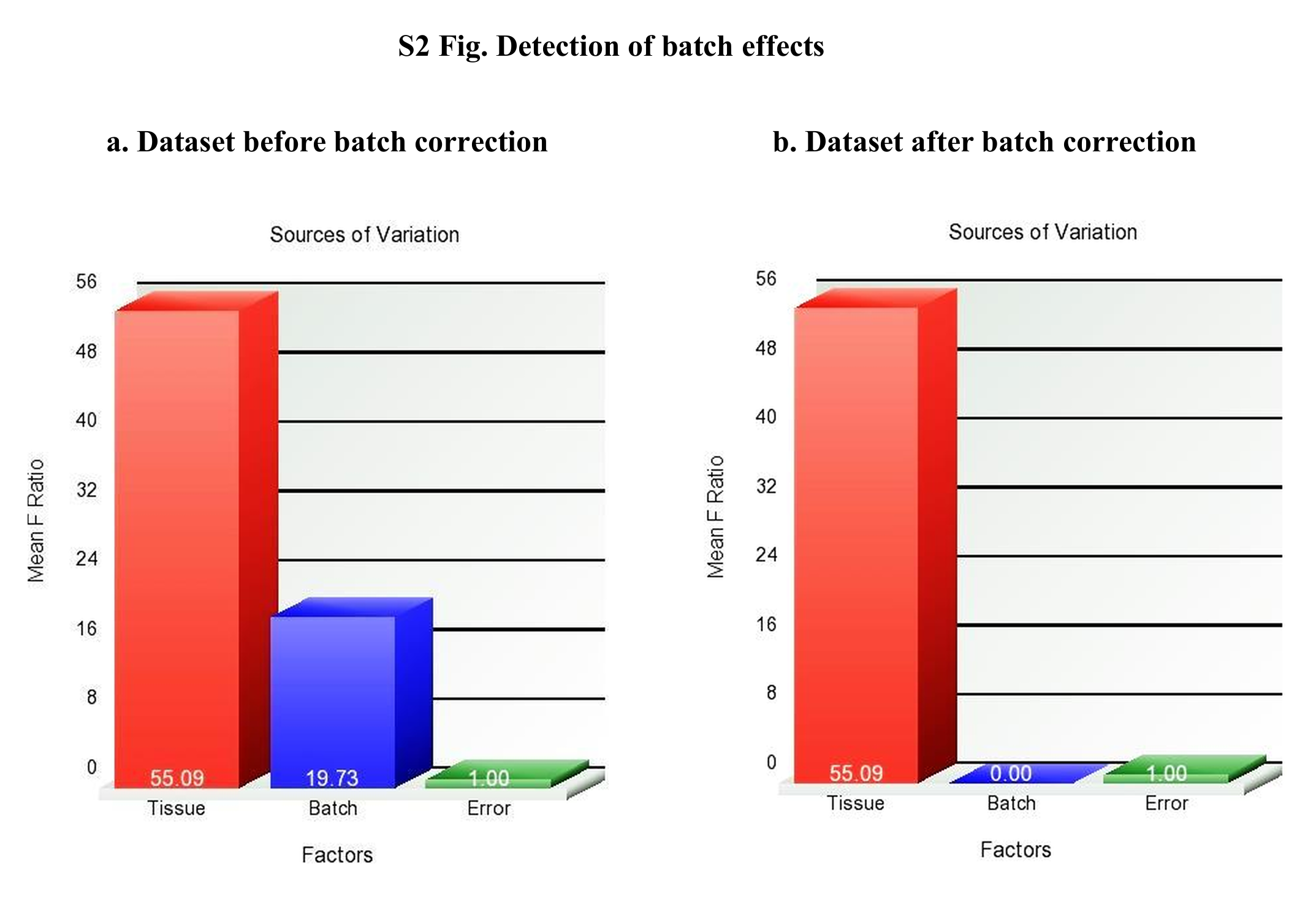

Supplement: S2 Fig — The raw counts of all 768 snoRNAs were RPKM normalized and corrected for batch effects. S2A Fig represents the data before batch effects correction (Mean F ratio of batch = 19.73) and S2B Fig represents the data after batch effects correction (Mean F ratio of batch = 0). The factor ‘tissue’ represents biological variation arising from normal and tumor tissues; hence was not appropriate to correct for. (TIF) [file pone.0162622.s002.tif]

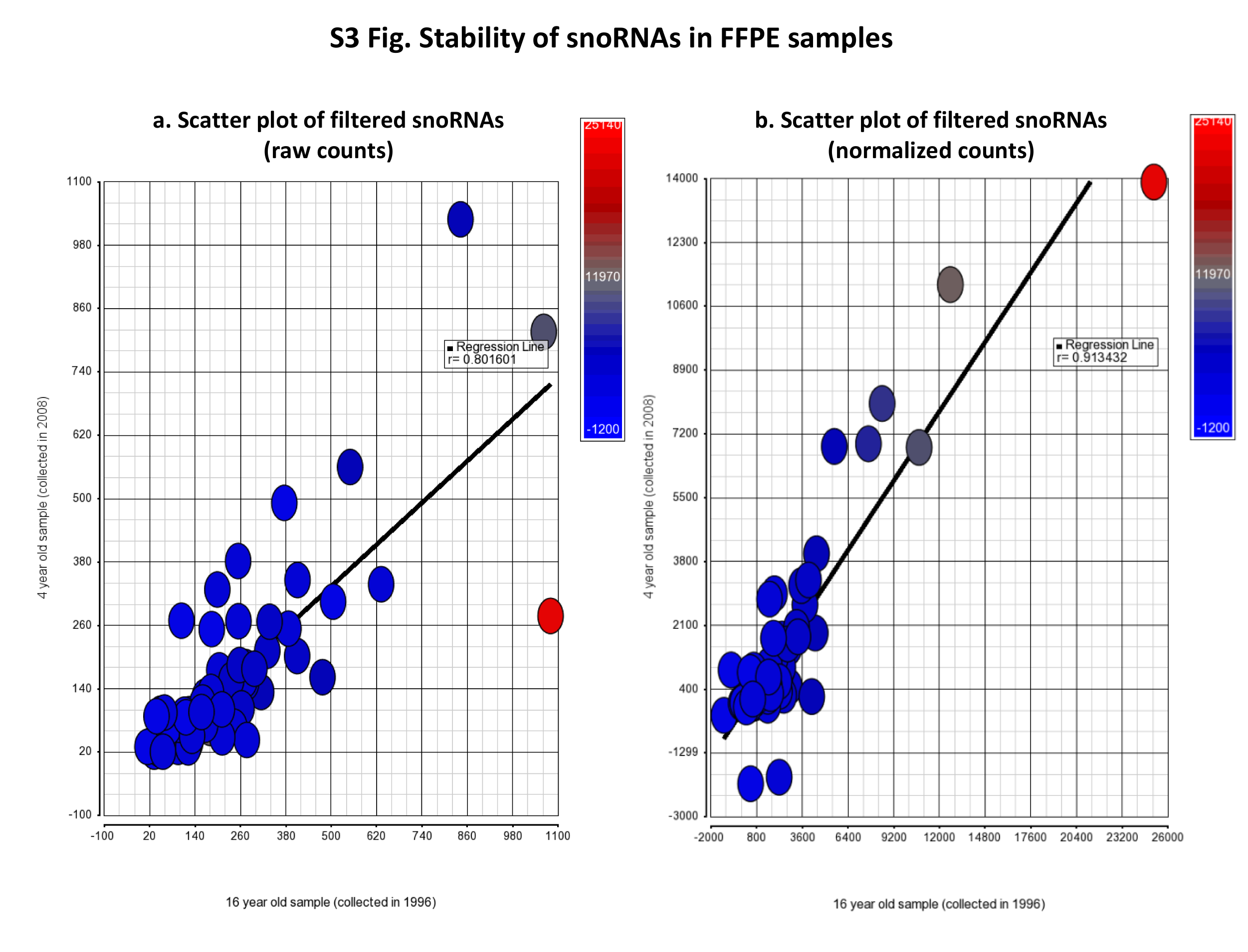

Supplement: S3 Fig — Scatter plots of 88 snoRNAs detected from a 16 year old sample (collected in 1996) and a 4 year old sample (collected in 2008). Correlation coefficients ≥ 0.8 from raw counts (a) and > 0.9 from batch adjusted normalized counts (b) indicate that the snoRNAs are stable in FFPE samples. (TIF) [file pone.0162622.s003.tif]
