# Supplementary material for: Profiling of Small Nucleolar RNAs by Next Generation Sequencing: Potential New Players for Breast Cancer Prognosis
Source: PLoS One. 2016 Sep 15;11(9):e0162622. doi: 10.1371/journal.pone.0162622 (PMC5025248; doi:10.1371/journal.pone.0162622)
Supplement: S2 Table — snoRNAs filtered for read counts in the CC approach were subjected to one-way ANOVA test to identify differentially expressed snoRNAs with fold change > 2.0 and FDR cut off ≤ 0.05. Forty snoRNAs were differentially expressed; 9 showed up-regulation and 31 showed down-regulation in tumors, relative to normal tissues. (PDF) [file pone.0162622.s005.pdf]

S2 Table. List of 40 differentially expressed snoRNAs

snoRNA ID	Fold Change	Direction of fold change	FDR value
SNORA45-201	3.92	Up-regulated in tumor	7.38E-08
SNORA31-001	53.00	Up-regulated in tumor	4.08E-06
SCARNA4-201	22.00	Up-regulated in tumor	4.99E-06
SNORD101-201	23.99	Up-regulated in tumor	8.77E-06
SNORA64-201	5.43	Up-regulated in tumor	9.95E-05
SNORA48-201	2.90	Up-regulated in tumor	6.43E-04
SNORD37-201	3.82	Up-regulated in tumor	1.42E-03
SNORD1B-201	5.08	Up-regulated in tumor	1.79E-03
SNORA65-201	3.24	Up-regulated in tumor	7.19E-03
SNORD61-201	-7.90	Down-regulated in tumor	2.05E-34
SNORD110-201	-24.22	Down-regulated in tumor	2.05E-34
SNORD18A-201	-5.90	Down-regulated in tumor	3.10E-34
SNORD68-201	-10.77	Down-regulated in tumor	4.13E-32
SNORD46-201	-7.38	Down-regulated in tumor	8.18E-32
SNORD89-201	-4.07	Down-regulated in tumor	4.08E-27
SNORD59A-201	-4.36	Down-regulated in tumor	4.60E-27
SNORD104-201	-4.37	Down-regulated in tumor	2.32E-26
SNORD5-201	-7.17	Down-regulated in tumor	7.46E-26
SNORD10-201	-4.06	Down-regulated in tumor	7.71E-26
SNORD99-201	-2.97	Down-regulated in tumor	3.45E-20
SNORA7B-201	-2.96	Down-regulated in tumor	5.63E-19
SNORD20-201	-3.52	Down-regulated in tumor	1.15E-17
SNORD119-201	-2.95	Down-regulated in tumor	1.27E-16
SNORD13-201	-3.10	Down-regulated in tumor	3.12E-16
SNORD102-201	-2.97	Down-regulated in tumor	1.59E-14
SNORD34-201	-3.18	Down-regulated in tumor	2.85E-14
SNORD63-201	-3.80	Down-regulated in tumor	6.56E-13
SNORD58A-201	-2.90	Down-regulated in tumor	1.55E-12
SNORD58B-201	-2.34	Down-regulated in tumor	3.19E-12
SNORD12C-201	-3.32	Down-regulated in tumor	3.86E-12
SNORD52-201	-3.95	Down-regulated in tumor	1.30E-11
SNORD111B-201	-2.83	Down-regulated in tumor	1.04E-09
SNORD15B-201	-2.83	Down-regulated in tumor	1.54E-09
SNORD71-201	-2.10	Down-regulated in tumor	1.56E-09
SNORA7A-201	-2.15	Down-regulated in tumor	4.07E-08
SNORD42A-201	-2.40	Down-regulated in tumor	1.11E-07

SNORD95-201	-2.43	Down-regulated in tumor	1.69E-07
SNORD58C-201	-2.40	Down-regulated in tumor	4.07E-06
SNORD84-201	-2.24	Down-regulated in tumor	2.35E-05
SNORD17-201	-2.12	Down-regulated in tumor	2.58E-05

FDR = False Discovery rate
