# Supplementary material for: Profiling of Small Nucleolar RNAs by Next Generation Sequencing: Potential New Players for Breast Cancer Prognosis
Source: PLoS One. 2016 Sep 15;11(9):e0162622. doi: 10.1371/journal.pone.0162622 (PMC5025248; doi:10.1371/journal.pone.0162622)
Supplement: S3 Table — In the CO approach, twelve and ten snoRNAs were identified for OS and RFS, respectively with permutation p-value ≤ 0.1. The snoRNAs identified in the CO approach encompassed all the snoRNAs identified in the CC approach for both OS (n = 5) and RFS (n = 4) and are highlighted in red. (PDF) [file pone.0162622.s006.pdf]

S3 Table. List of snoRNAs with prognostic relevance for breast cancer

snoRNAs significant for OS	snoRNAs significant for RFS
SNORA7A	SNORA7A
SNORA7B	SNORA7B
SNORD100	SNORD100
SNORD104	SNORD105
SNORD105	SNORD14E
SNORD14E	SNORD46
SNORD46	SNORD82
SNORD82	SNORD89
SNORD84	SNORD92
SNORD85	SNORD94
SNORD92	
SNORD94	
