# Supplementary material for: Profiling of Small Nucleolar RNAs by Next Generation Sequencing: Potential New Players for Breast Cancer Prognosis
Source: PLoS One. 2016 Sep 15;11(9):e0162622. doi: 10.1371/journal.pone.0162622 (PMC5025248; doi:10.1371/journal.pone.0162622)
Supplement: S5 Table — (PDF) [file pone.0162622.s008.pdf]

S5 Table. Gene ontology terms associated with genes targeted by piRNAs within snoRNAs

Gene Ontology term	Genes involved	piRNAs targeting genes
Positive regulation of cell adhesion	ARHGAP6, CNTNAP3, CNTN2	hsa_piR_018780, hsa_piR_004531
Response to hormone	AVPR1A, BCHE, TXNIP	hsa_piR_000045, hsa_piR_018780 hsa_piR_004531
Regulation of apoptotic process	BCL6, ALB, SFRP1, TXNIP, FOSL1, GRM4, PRAME	hsa_piR_000045, hsa_piR_018780, hsa_piR_004531, hsa_piR_017033, hsa_piR_001078, hsa_piR_019102
Cell communication	BCL6, AVPR1A, BCHE	hsa_piR_000045, hsa_piR_018780
Regulation of cell proliferation	BCL6, SMAD2, ADRA2A, AVPR1A, ASPH, TXNIP, FOSL1, PRAME, TNFSF4	hsa_piR_000045, hsa_piR_018780, hsa_piR_004531, hsa_piR_017033, hsa_piR_019102
Transcription	BCL6, SMAD2, TXNIP, TFAP2C, ZNF462, BRIP1, FOSL1, HOXC13, TNFSF4	hsa_piR_000045, hsa_piR_018780, hsa_piR_004531, hsa_piR_017184, hsa_piR_017033, hsa_piR_019676
G-protein coupled receptor signaling pathway	GPR26, CENPI, DGKH, GRM4, CGA	hsa_piR_001078, hsa_piR_019676
Signal transduction	RSPO3, SMAD2, ADRA2A, ANGPTL1, AVPR1A, SFRP1, TXNIP, ADAMDEC1, GPR26, CENPI, DGKH, GRM4, CGA	hsa_piR_000045, hsa_piR_018780, hsa_piR_004531, hsa_piR_019676, hsa_piR_001078
Cell-cell signaling	SMAD2, SLC6A2, TFAP2C	hsa_piR_018780, hsa_piR_004531, hsa_piR_000045
